# Capacity of antiretroviral therapy sites for managing NCDs in people living with HIV in Zimbabwe

**DOI:** 10.4102/sajhivmed.v21i1.1113

**Published:** 2020-09-04

**Authors:** Laston Gonah, Indres Moodley, Khumbulani Hlongwana

**Affiliations:** 1 Health Outcomes Research Unit, Discipline of Public Health Medicine, School of Nursing and Public Health, College of Health Sciences, University of KwaZulu-Natal, Durban, South Africa

**Keywords:** hypertension, diabetes mellitus, HIV-NCD comorbidity, NCD management protocols, NCD screening and treatment

## Abstract

**Background:**

There are marked inconsistencies in prevalence data for human immunodeficiency virus and non-communicable disease (HIV-NCD) comorbidity in Zimbabwe.

**Objectives:**

To explain these discrepancies, we investigated the capacity of antiretroviral therapy (ART) sites in managing hypertension (HTN) and diabetes mellitus (DM) in people living with HIV (PLWH) in Gweru district, Zimbabwe.

**Method:**

This was a qualitative research design in which key informant interviews were conducted with eight health managers, and 12 focus group discussions (FGDs) were conducted with 72 PLWH concurrently diagnosed with HTN and/or DM. Thematic data analysis was performed in NVivo version 12®.

**Results:**

Routine screening for HTN and targeted screening for DM were often interrupted by dysfunctional machines and intermittent supply of necessary consumables, impacting negatively on the capacity of the sites to monitor and screen for the NCDs. Erratic hypertensive and diabetic medication availability at study sites were also reported, forcing patients to turn to other treatment options (medication rationing or overdose or sharing, use of home remedies and traditional medicines, and reliance on faith and traditional healers).

**Conclusion:**

Findings demonstrate that the quality of observed incidence and prevalence data for HTN and DM in LMICs is a function of the capacity of health centres to screen for NCDs. Given the ageing population of PLWH in sub-Saharan Africa, coupled with increasing trends in the prevalence of NCDs in HIV-infected people, HIV programmes have not evolved with the changing needs of PLWH. Attention to the holistic management of PLWH is long overdue.

## Introduction

Unprecedented global improvement in enabling access to antiretroviral therapy (ART) by people living with human immunodeficiency virus (PLWH), contributing to increased viral suppression and increased survival in PLWH, is well documented.^1^ By the end of 2018, more than 61% of the 37.9 million PLWH, globally, were accessing ART, which is a substantial increase from 45% in 2010.^1^ Over the same period, acquired immunodeficiency syndrome (AIDS)-related deaths decreased by about 45%,^1^ thereby demonstrating the significant contribution of ART to increased survival in PLWH.

While there is empirical evidence showing the positive contribution of ART to improved survival in PLWH, new threats associated with non-communicable diseases (NCDs) co-existing with human immunodeficiency virus (HIV) in PLWH have emerged.^2^ This co-existence is likely to impact negatively on the gains already achieved in controlling HIV, especially in many low- to middle-income countries (LMICs) where the majority of PLWH on ART live and where health care systems are known to be fragile.^2^ The real effects of NCDs co-existence with HIV in PLWH in many settings may be undermined by the lack of accurate data to understand this phenomenon fully. For example, there are marked inconsistencies in the Zimbabwean prevalence data for HIV-NCD comorbidity.^3,4,5^ These epidemiological discrepancies can be best explained by studying the capacity of ART sites to manage NCDs in PLWH, that is, the screening, diagnosis, treatment and data management. Limited capacity to screen and diagnose diseases or conditions may compromise the quality of the prevalence data generated.

In Zimbabwe, more robust and consistent evidence exists for the management of tuberculosis (TB) – a well-known leading cause of death in PLWH.^6,7^ Contrary to the worldwide situation with TB, which is well integrated into HIV care allowing active TB case finding and better case management, there is limited evidence on how HIV-NCD comorbidity is being managed in Zimbabwe.^6,7^

Against this background of discrepancies as reported in the current literature, a qualitative study was conducted in Gweru district of Zimbabwe among healthcare workers and PLWH. The study sought to explore the treatment of hypertension (HTN) and diabetes mellitus (DM) in PLWH attending ART sites and to assess the current capacity of these sites to manage HTN and DM. We also investigated the challenges faced by PLWH diagnosed with HTN and/or DM in accessing care at the study sites and their ways of coping up with the challenges.

Gweru district is one of the unique districts in Zimbabwe in the sense that it comprises both urban and rural populations, a phenomenon different from the other major metropolitan provinces such as Harare and Bulawayo, which consist of urban populations in their entirety. Gweru district is in the Midlands Province, where the prevalence of HIV is relatively higher than some other provinces.^8^

## Methods

### Design and setting

This was an exploratory, qualitative study utilising data collected from key informant interviews and focus-group discussions (FGDs). Six high-volume public ART sites situated at primary healthcare clinics were selected for the study. These were those with the highest number of PLWH (≥1000 PLWH) registered for ART in the Gweru district. Four sites were situated in the urban part. The remaining two sites were in the rural sector.

### Sampling and data collection

Key-informant interviews were conducted with the District Medical Officer (DMO), the City Health Director and a Nurse-in-Charge of each of the six ART sites. Each of the key informants had special expertise and experience in managing NCDs and HIV in the district. A total of eight key-informant interviews were conducted in the English language, using a key-informant interview guide. The guide was designed to assess how HTN and DM were being managed at the ART sites and to assess the capacity of the sites to manage PLWH with these comorbidities. Each interview lasted an average of 50 minutes. Where necessary, observations were conducted with key informants to corroborate data. The English language was used given its status as the official language of the workplace.

Focus group discussions were conducted among PLWH with HTN and/or DM. A standard FGD guide was used to elicit care perspectives on comorbid disease care and experiences and/or challenges encountered while accessing care at the ART centres. We also enquired about the coping strategies employed in dealing with the identified challenges. Purposive sampling criteria were used to identify the FGD participants.^9^ These were ART-registered PLWH who had HTN and/or DM and had reported for care at least once a month for the previous 3 months prior to the study – confirmed by patient site records. The average time taken to complete each FGD was 65 minutes. The data collection tools, namely the interview guide and the FGD guide, were validated by (1) having them separately checked for appropriateness by three doctorate holders, who had particular experience in health sciences research, (2) pilot testing the interview guide among health managers from another district and (3) pilot testing the FGD guide among other potential participants who were later excluded from the final study. The data collection tools were modified according to pilot test responses before being used in the final data collection.

Written informed consent was sought from potential participants. Age (30–49; and >50 years), gender (male and female) and geographical background (rural and urban) were considered as key characteristics that could influence free and natural discussion. Therefore, homogeneity of characteristics (similar age group, gender and geographical background) within FGDs was ensured to promote unrestricted group member participation. Heterogeneity of participants in terms of age, gender and geographic background was ensured across all FGDs to capture diverse perspectives. Each FGD was composed of six participants. All FGDs were conducted in local languages (Shona and/or Ndebele) using an FGD guide. Translation of FGD data to the English language was performed in consultation with three experts of English, Shona and Ndebele languages during data transcription.

Both interviews and FGDs were conducted by the principal investigator who has some training in qualitative research methods. Furthermore, the co-author who guided the research process had experience as a qualitative researcher. Notes were taken during interviews, and FGDs, whereas session summaries and debriefing notes were produced from each interview and FGD conducted. All interview and FGD sessions were recorded using two audio recorders. Analysis and data collection were iterative, with interviews and FGDs continuing until code saturation, that is, until no new codes were emerging.^10^ When the last Nurse-in-Charge was interviewed at the sixth clinic, it was found that no new themes or information had emerged and that code saturation had been reached with the eight interviews. No further interviews were conducted with additional employees. As for FGDs, 12 FGDs (six participants per one FGD) were conducted before reaching the saturation point.

### Data management and analyses

Data from the interviews and FGDs were stored as audio-recordings, interview notes, FGD notes, session summaries and debriefing notes. Audiotapes were transcribed verbatim onto a word-processing programme. Analyst triangulation was achieved through the two researchers independently analysing all emerging codes and issues across interviews and FGDs, and later discussing them to ensure that the results were grounded on data rather than the researchers’ perceptions. Thematic analysis of qualitative data was performed in NVivo version 12®, involving familiarisation with the data, generation of initial codes, searching of themes within the codes, reviewing, defining and naming themes. Findings were presented according to emerging themes, using verbatim quotes to illustrate and support the analysis.

## Findings

### Participant characteristics

A total of eight interviews were conducted with key informants, whereas 12 FGDs were conducted with PLWH concurrently diagnosed with HTN and/or DM (Table 1). The basic requirement for a Nurse-in-Charge is a diploma, whereas that for a DMO and/or City Health Director is a degree.

**TABLE 1:** Interview and focus group discussion participants’ characteristics.

Participants’ characteristic	Number	%
**Key informant interviews (*n* = 8)**		
**Age**		
30–49 years	7	87.5
> 50 years	1	12.5
**Sex**		
Male	3	37.5
Female	5	62.5
**Highest level of education**		
Diploma	4	50
Degree	4	50
**Focus group discussions (*n* = 72)**		
**Age**		
30–49 years	36	50
> 50 years	36	50
**Sex**		
Male	24	33.3
Female	48	66.7
**Highest level of education**		
Secondary	45	62.5
Tertiary	27	37.5

### Findings from key informant interviews

Key themes that emerged from key-informant interviews pointed on issues to do with how HTN and DM were being managed, and the capacity of ART sites to screen, diagnose and treat HTN and DM. The themes and subthemes emerging from each FGD are summarised in Table 2.

**TABLE 2:** Saturation grid of themes and subthemes emerging from key informant interviews.

Themes and subthemes	Interview number							
	1	2	3	4	5	6	7	8
**Management of HTN and DM**								
• Routine screening of HTN on every consultation	x	x	x	x	x	x	x	x
• Targeted screening of DM based on signs and symptoms	x	x	x	x	x	x	x	x
**Capacity to screen for HTN and DM**								
• Adequate number of staff capable of screening for HTN and DM in place	x	x	x	x	x	x	x	x
• Have at least one BP and RBS machine	x	x	x	x	x	x	x	x
• Reported dysfunctional BP machine	x	x	x	x	x	x	x	x
• Reported battery or power challenges for BP machine	x	x	x	x	x	x	x	x
• No glucostrips in stock	x	x	x	x	x	x	x	x
• Inadequate funding for NCD management	x	x	x	x	x	x	x	x
**Capacity to treat HTN and DM**								
• HTN drugs out of stock	x	x	x	x	x	x	x	x
• DM drugs out of stock	x	x	x	x	x	x	x	x
• HCT sometimes available	x	x	x	x	x	x	x	x

DM, diabetes mellitus; HCT, hydrochlorothiazide; HTN, hypertension; NCD, non-communicable diseases.

### Management of hypertension and diabetes mellitus

Findings from key informants consistently concurred on how screening services for HTN and DM were being offered:

‘Ministry [*of Health*] regulations require vital observations (including blood pressure, pulse rate, weight, and temperature measurements) to be done at every consultation as part of routine screening, to all patients regardless of HIV status …. However, the regulations require targeted screening for diabetes mellitus where random blood sugar (RBS) measurements are done only when a patient presents with signs and symptoms suggestive of diabetes mellitus.’ (Male, 45 years old, Health Executive)

It was found that both BP and RBS measurements were being offered as part of the consultation process at all ART sites, where HIV-negative people pay a consultation fee equivalent to USD$1, and PLWH do not pay any consultation fee in urban sites. In rural sites, both HIV negative and PLWH do not pay any consultation fee.

Nurses-in-Charge for all the study sites consistently mentioned existence of Community ART Review Groups (CARGs):

‘There are CARGs, of 7–10 PLWH per group. Through CARGS, only one group member collects medication on behalf of other group members, thereby reducing transport cost for collection of ART medication. This is useful especially when the patient does not require other services like viral load measurements, HTN/DM screening or other consultation services.’ (Female, Nurse-in-Charge, with over 10 years’ experience)

### The capacity of antiretroviral therapy sites to screen, diagnose and treat non-communicable diseases

With regard to the capacity of ART sites to manage HTN and DM, the availability of human, financial and material (equipment and drugs) resources for screening, diagnosis and treatment was assessed at each ART centre.

### Human resources

All key informants confirmed that the general nurses present at all ART sites were capable of screening for HTN and DM, even though they lacked at least one speciality, namely training in endocrinology. However, the lack of equipment remained a leading challenge:

‘All the ART sites are manned by nurses who can screen for hypertension and diabetes mellitus if they have the required equipment. However, we do not have specialised endocrinology nurses for NCD care at any of ART sites in the district.’ (Male, District Health Executive, with over 6 years’ experience)

### Material resources (equipment and drugs)

Screening for HTN requires a blood pressure (BP) machine, whereas screening for DM requires a random blood sugar (RBS) machine and glucostrips. Some BP machines used in the study sites were battery-powered, while others were electric-powered. Random blood sugar machines at all study sites were battery-powered. All sites had at least one BP and RBS machine.

Interview findings from all study sites consistently found that availability of screening services for HTN and DM was generally limited and periodically interrupted by various challenges. These included running out of machine batteries without timely replacement for HTN screening and frequent electrical power cuts that affected the use of electric-powered BP machines:

‘…. *Y*es, we have a BP machine here. But we have not been using it for the past 2 months, because it doesn’t have batteries.’ (Female, Nurse-in-Charge, at a rural ART site)

Another reported challenge was the dysfunctional BP machines caused by the machine breakdowns. In three (two urban and one rural site) of the six study sites, the availability of only one BP machine was insufficient to service all clinic departments, including the outpatients and the maternity departments. For targeted screening for DM, all the sites reported that the service was generally not available, largely because of non-availability of glucostrips:

‘The major challenge affecting screening for diabetes mellitus is the unavailability of glucostrips. There is a general shortage of glucostrips in our health centres, so patients might not have their blood glucose levels checked when they require the service …. For hypertension screening, some health centres do not have functional BP machines due to issues like machine breakdowns, electricity power cuts, and unavailability of batteries, among other reasons.’ (Male, District Health Executive, with 5 years’ experience)

Concerning the availability of drugs for HTN and DM, the interview findings revealed the partial availability of HTN drugs and the general non-availability of DM drugs. There was a convergence of views from all the study sites in that some hypertensive patients usually on hydrochlorothiazide (HCT) drug sometimes had access to the drug. However, hypertensive patients may need more than one type of drug, where some combinations might not include HCT. None of the key informants reported having any diabetic medication in stock:

‘Stock-outs for hypertensive and diabetic medication are generally common due to limited funding for NCDs. Most patients usually get HCT from most of our health centres.’ (Male, Health Executive, with over 6 years’ experience)

While patients are unable to access screening services or medication for either HTN or DM or both, they are usually referred to private pharmacies where they must pay transportation costs, and the cost of the screening service and/or the medication costs:

‘If they can’t get medication here, we write them a prescription to purchase in town [*in private pharmacies*] if they have the funds.’ (Male, Nurse-in-Charge, with over 15 years’ experience at an urban ART site)

## Financial resources

Responses pointed to limited financial resources for NCD management. Interviewees consistently reported that funding was inadequate to meet the needs of people at risk of or living with NCDs:

‘The main source of funds for NCD management is the government of Zimbabwe (GoZ). Unlike the integrated HIV and TB care programmes, which receive additional funding from Non-governmental organizations (NGOs) like the Global Fund and USAID, NCD programmes are funded largely by the government, where the funding is usually limited. Funding is required for maintenance of equipment, procurement of equipment, drugs and medical sundries, … even training and re-training of our healthcare workers on NCD management.’ (Male, Health Executive, with 5 years’ experience)

### Focus group discussion findings from patients

A total of 12 FGDs (consisting of six participants each) were conducted. More FGDs (*n* = 8) were conducted among female participants than among male participants (*n* = 4). Other characteristics of FGD participants are shown in Table 3.

**TABLE 3:** Characteristics of focus group discussions of people living with human immunodeficiency virus and comorbid non-communicable diseases (*n* = 72).†

Characteristics	Urban participants				Rural participants			
	Male		Female		Male		Female	
Age (years)	30–49	≥ 50	30–49	≥ 50	30–49	≥ 50	30–49	≥ 50
Number of FGDs	1	1	2	2	1	1	2	2

† , Each focus group discussion (FDG) consisted of six participants, translating to a total of 72 participants for the 12 FGDs.

### Challenges faced by patients in accessing non-communicable diseases care and related coping mechanisms

The analysis of data generated from the FGDs revealed the non-availability of BP and RBS measurement services, the non-availability of HTN and DM medication at ART sites, the unaffordable cost of medication at private pharmacies and the challenges of transportation costs when seeking medical care or medication, as common challenges faced by patients in accessing care for HTN and DM. The saturation grid for themes and subthemes emerging from the FGDs is presented in Table 4. The FGD findings at both urban and rural sites were consistent with those from the key informant interviews.

**TABLE 4:** Saturation grid of themes and subthemes emerging from focus group discussions.

Themes and subthemes	Focus group discussion number											
	1	2	3	4	5	6	7	8	9	10	11	12
**Challenges in accessing healthcare**												
• Non-availability of HTN screening	x	x	x	x	x	x	x	x	x	x	x	x
• Non-availability of RBS screening	x	x	x	x	x	x	x	x	x	x	x	x
• Non-availability of HTN medication	x	x	x	x	x	x	x	x	x	x	x	x
• Non-availability of DM medication	x	x	x	x	x	x	x	x	x	x	x	x
• Unaffordable cost of medication in private pharmacies	x	x	x	x	x	x	x	x	x	x	x	x
• Challenges with transport costs to seek medical care or medication	x	x	x	x	x	x	x	x	x	x	x	x
**Coping strategies or mechanisms**												
• Taking multiple drugs to compensate missed doses	x	x	x	x	x	x	x	x	x	x	x	x
• Sharing medication with colleagues	x	x	x	x	x	x	x	x	x	x	x	x
• Rationing of medication	x	x	x	x	x	x	x	x	x	x	x	x
• Use of home remedies	x	x	x	x	x	x	x	x	x	x	x	x
• Use of traditional herbs	x	x	x	x	x	x	x	x	x	x	x	x
• Consultation of traditional or faith healers	x	x	x	x	x	x	x	x	x	x	x	x

DM, diabetes mellitus; HTN, hypertension; RBS, random blood sugar.

### Challenges in accessing healthcare

There was consensus across all FGDs that BP and RBS measurement services were not always available at all study sites. Participants pointed to repeated power cuts affecting the electric-powered BP machines, the battery-powered machines running out of batteries or service non-availability because of use of the machine in other clinic departments:

‘…. As for me, my BP was only checked once this year. Most of the time I come here there will be no electricity so the BP machine will not be working. Sometimes you come here, and you are told the machine is being used in the maternity ward. As for sugar [*diabetes mellitus*], I don’t even know whether the machine is here or not because I have never received the service here.’ (Female, 55 years old, participant with HTN & DM)

The other commonly emerging subtheme from all FGDs was of medication availability for HTN and DM. Participants from all the ART sites confirmed that HCT is the only medication for HTN, which some might access. However, some hypertensive patients are not prescribed HCT, while others take more than one variety of medicine, none of which can be accessed at the clinic. None of the patients from all the 12 FGDs reported to have accessed DM medication:

‘Sometimes they give HCT, but it is not everyone who gets it. Again, some of us do not take HCT, I take 4 other drugs for hypertension. For sugar [*diabetes mellitus*], I have never received medication from this clinic.’ (Male, 62 years old, participant with HTN and DM)

When they cannot get medication from the clinic, participants reported getting prescriptions to purchase the required medication from private pharmacies, where the main challenge is the high cost of medication and transportation:

‘They give a prescription for you to buy in the [*private*] pharmacies in town. There drugs are very expensive and some of us cannot even afford them. To go there you need transport fare again. It’s really not easy.’ (Female, 68 years old, participant with diabetes mellitus)

#### Coping mechanisms

Common coping subthemes included taking multiple doses to compensate for missed doses, sharing and rationing the medication. Furthermore, the use of home remedies, traditional herbs and consultation of traditional and faith healers emerged as coping strategies to deal with the identified challenges in managing HTN and DM.

It emerged that when patients do not have enough medication to last the whole month, there was a general consensus that they ration the medication by only taking medication when they experience serious illness. Also reported was that patients often took multiple doses of HCT to complement the missing medication, which would not have been purchased, or when they perceive symptoms to be associated with their hypertension:

‘You go to the clinic and they give you only HCT. Apart from HCT, I need 3 more types of BP drugs. So when it gets serious, I take 4 HCT pills at once to relieve the pain, and it works.’ (Male, 57 years old, participant diagnosed with HTN 10 years ago)

Others reported borrowing medications when they run out of medication:

‘Last time I fell seriously ill and I could feel I was dying. I did not have the medication and transport fare to go to the clinic either. I ended up borrowing the medication from my neighbour, that is how I got saved. We always help each other that way.’ (Female, 68 years old, participant diagnosed with HTN and DM)

Concerning home remedies, the use of ginger, garlic, *cassia abbreviata* (*murumanyama* in local *Shona* language) and smashed avocado seeds commonly reported remedies used to manage HTN. Diabetic patients often used ginger, boiled black-jack and diet monitoring as remedies or alternatives for managing DM when they could not access medication:

‘I rely a lot on those [*remedies*]… especially garlic, *murumanyama* [*cassia abbreviata*] and [*smashed*] avocado seeds to manage my [*high*] BP … ginger and boiled black-jack also works for sugar [*DM*]… What can we do?… I can’t afford these [*allopathic*] medicines.’ (Female, 57 years old, participant diagnosed with HTN and DM)

Patients also reported that they relied on faith and traditional healers for the management of HTN and DM. Most participants across all FGDs mentioned that faith and traditional healers play an important role in managing their HTN and DM. Although all generally believed that allopathic medicines were more effective, they reported resorting to faith and traditional healers because they were cheaper alternatives. Participants also reported that these faith and traditional healers provided ‘spiritual-uplifting’ in the absence of medical drugs:

‘I know that [*prescribed*] drugs are effective… but one needs money to buy them. Prophets, traditional healers … and home remedies are cheaper or even free, … so I can’t watch myself die when these options are there.’ (Male, 61 years old, participant with DM)

In some worst-case scenario, some reported avoiding formal healthcare for fear of what they called ‘unnecessary costs’:

‘I do not even go to the [*provincial*] hospital, because when I go there, sometimes they tell me my [*blood*] sugar [*level*] is very high and give me bed-rest, but not the medication. The next day, they give me a huge bill to pay for the bed-rest when I did not have medication. Better I rest at home.’ (Female, 48 years old, participant with DM)

## Discussion

This study assessed the capacity of ART sites to manage HTN and DM in PLWH, from the perspectives and experiences of key healthcare workers and affected patients in the Gweru district of Zimbabwe. Also assessed were patients’ experiences on the challenges they face in accessing care and the coping mechanisms they employ in dealing with the challenges. Several important findings emerged: (1) ART sites had limited capacity to screen for HTN and DM in PLWH, together with non-availability of HTN and DM medication, because of limited funding available for NCDs care; (2) PLWH who have HTN and/or DM are deterred from accessing healthcare for HTN and DM at public ART sites, mainly because of frequent unavailability of screening and treatment services and the high costs of medication in private pharmacies; (3) coping strategies employed in addressing encountered challenges include taking multiple doses to compensate for missed doses, sharing medication with others, medication rationing, the use of home remedies and traditional herbs, and the consultation of traditional or faith healers.

In this study, HTN screening was found to be integrated into routine screening as part of vital measurements which should be performed at every clinic consultation regardless of HIV status. In principle, this is a strategic arrangement to increase chances of hypertensive case finding as well as reducing stigma associated with screening only one group of patients.^7^ For DM, the Zimbabwean Ministry of Health’s regulations require targeted screening where RBS measurements are taken from patients presenting with signs and symptoms suggestive of high blood sugar levels.^7^ While the protocols and cost of screening for HTN and DM could promote active case finding, in this study, effective implementation of the process was threatened by non-functional equipment secondary to limited funds. Interrupted provision of screening services can negatively affect diagnosis of new cases and ultimately result in lower than actual incidence and prevalence rates being observed for HTN and DM, as has been found in other studies.^11^

Erratic availability of HTN and DM medication at ART sites was reported as another common challenge faced by patients, as observed in other similar studies.^4,12,13^ For the majority of patients who could not afford medication from private pharmacies, alternative approaches were taken. The reported options included medication rationing or sharing or overdose, for example, multiple doses to compensate when the medication became available and the use of home remedies and herbs. These options do not offer effective treatment for HTN and DM because they are not regulated, nor is their effectiveness subjected to empirical research.^7^ If HTN and DM are not managed well in PLWH, the survival and wellness gains of ART will be sacrificed.

The unprecedented scale-up of ART in sub-Saharan Africa (SSA) has resulted in more PLWH achieving virologic suppression and increased survival.^14^ Therefore, the incidence of diseases associated with ageing such as HIV-HTN, DM, chronic kidney and heart disease will also increase.^2,14,15,16,17,18^ A study conducted in Malawi revealed that the lack of screening equipment and services, and medication stock-outs were frequent challenges to the care of PLWH and HTN/DM.^19^ Other studies conducted in SSA focused more on the feasibility of integrating HIV/AIDS programme with NCD care, and key findings point to the existence of challenges in screening for HTN and DM compared to those for HIV/AIDS care.^17^ There is limited research into the capacity of SSA to manage the future challenges faced by the long-term survivors of HIV.

## Strengths and limitations

Use of purposive sampling based on set criteria to identify participants; involvement of more than one experienced qualitative researcher to reduce the chances of subjective bias; and attempts to attain code saturation in data collection represent methodological strengths of the study.

The weakness of the study was the use of cross-sectional study design that prevents long-term follow-up of participants and the absence of ‘hard’ clinical end points – actual BP, glucose monitoring and viral load suppression of the study cohort over time – to support the contention that the care of this group of Zimbabwean patients is being compromised.

## Conclusion

Findings from this study demonstrate that the observed incidence and prevalence of HTN and DM among PLWH in Gweru district is a function of the capacity of its healthcare system to screen for and adequately manage NCDs. Given the ageing population of PLWH in SSA, coupled with the growing prevalence of NCDs in this group, HIV programmes require strengthening. While the social and economic problems of Zimbabwe are dire, this study gives voice to some of its PLWH. We feel that this is a voice that others need to hear too.
